# Newborn colonization and antibiotic susceptibility patterns of *Streptococcus agalactiae* at the University of Gondar Referral Hospital, Northwest Ethiopia

**DOI:** 10.1186/s12887-018-1350-1

**Published:** 2018-11-30

**Authors:** Mucheye Gizachew, Moges Tiruneh, Feleke Moges, Mulat Adefris, Zemene Tigabu, Belay Tessema

**Affiliations:** 1 Department of Medical Microbiology, School of Biomedical and Laboratory Sciences, College of Medicine and Health Sciences, University of Gondar, P. O. Box 196, Gondar, Ethiopia; 2 Department of Gynecology and Obstetrics, School of Medicine, College of Medicine and Health Sciences, University of Gondar, P. O. Box 196, Gondar, Ethiopia; 3 Department of Pediatrics, School of Medicine, College of Medicine and Health Sciences, University of Gondar, P. O. Box 196, Gondar, Ethiopia

**Keywords:** Antibiotic susceptibility pattern, Colonization, Group B Streptococcus, Newborns

## Abstract

**Background:**

Group B *Streptococcus* (GBS) that asymptomatically colonizing the recto-vaginal area of women is the most important cause of neonatal colonization. There is paucity of evidence about newborn colonization with GBS in Ethiopia. Thus, this study was aimed to determine the prevalence of newborn colonization with GBS, antibiotic susceptibility patterns of the isolates and associated risk factors at the University of Gondar Referral Hospital in Northwest Ethiopia

**Methods:**

A prospective cross sectional study was conducted from December 2016 to November 2017. A total of 1,155 swabs from nasal, ear and umbilical areas of the newborns were collected from the 385 newborns. Identifications of the isolates and antibiotic susceptibility testing were done by using conventional methods.

**Results:**

Sixty two (16.1%, 95% CI: 12.2% - 20%) of the newborns were colonized by GBS. Seven percent of the total specimens were positive for GBS. The antibiotics susceptibility rates of GBS (average of the three body sites tested) were 95.1%, 89.6%, 88.9%, 85.7%, 85.3%, 81.3%, 76.9%, 76.1%, 73.8%, and 34.4% to ampicillin, penicillin, ciprofloxacin, chloramphenicol, vancomycin, azitromycin, erythromycin, clindamycin, ceftriaxone, and tetracycline, respectively. A multilogistic regression analyses were shown that the newborns that were from mothers whose education status was below tertiary level, and newborns from mothers who were: being employed, being nullipara and multigravida were at risk for colonization with GBS.

**Conclusion:**

Prevalence of neonatal colonization with GBS was higher than it was reported in three decades ago in Ethiopia. Ciprofloxacin, chloramphenicol, vancomycin and azithromycin were identified as the drug of choice next to ampicillin and penicillin.

**Electronic supplementary material:**

The online version of this article (10.1186/s12887-018-1350-1) contains supplementary material, which is available to authorized users.

## Background

The 2016 Ethiopian Demographic and Health Survey (EDHS) indicates that the overall mortality rate of under five children is 67/1000 live births, with the infant mortality rate of 48% (29% neonatal and 19% post-neonatal) deaths/1,000 live births. The estimate of child mortality is 20 deaths/1000 children surviving to 12 months of age [1]. Women in the Amhara National Regional State have the fertility rate of 4.2, and infant and maternal mortality rates of 76/1000 live births and 676/100,000, respectively [2]. Asymptomatic *Streptococcus agalactiae* (Group B Streptococcus, GBS) recto-vaginal colonization of women is assumed to be one of the contributing factors. It is the most significant pathogen, although little is known about its epidemiology and risk in resource limited countries [3]. Since neonatal infections cause a significant proportion of deaths in the first week of life, more data are needed about the burden of neonatal colonization [4].

Since 1960s, GBS has been identified as a major public health problem that causes perinatal morbidity and mortality. It also became the most prevalent causes of fatal infections in newborns [5–7]. The researchers estimated about 410,000 GBS cases and 147,000 stillbirths and infant deaths are estimated to occur every year. Despite containing 13% of the world's population, Africa had the highest burden with 54% cases and 65% of stillbirths and infant deaths [8]. GBS causes sepsis, pneumonia, and meningitis in neonates; bacteraemia, amnionitis, endometritis, and urinary tract infection in pregnant women [9–11]. The Global prevalence of GBS neonatal colonization rate ranged from 1.6% in Turkey [12] to 52.9% in Pakistan [13], and South Africa took the lion share among few African reports [14]. However, evidence on GBS colonization rate of newborns largely remains sparse in the African setting, particularly in Ethiopia.

Furthermore, provision of empiric treatment brings up antibiotic resistance and stewardship issues [8]. Reports from different countries revealed the reduced susceptibility to penicillin, and the increased rate of macrolide resistance GBS isolates for the last few decades [15]. A 2005-2007 Surveillance in Argentina showed the presence of GBS isolates resistance (in minimum inhibitory concentration; MIC range μg /L) to ciprofloxacin (32-64 μg/L), levofloxacin (16-32 μg/L), ofloxacin (32-64 μg/L), and norfloxacin (32-64 μg/L), and all were susceptible to penicillin (0.06 μg/L) (16). Of the 1160 GBS isolates in Australia, 6.4% demonstrated erythromycin resistance and 4.2% to clindamycin [16]. Another study in USA revealed that all the neonatal GBS were susceptible to penicillin, vancomycin, chloramphenicol, and cefotaxime. Its resistance rates to erythromycin was 20.2%, and 6.9% to clindamycin [17]. Another study in France revealed 38.2% erythromycin and 25.6% clindamycin resistance neonatal GBS [18]. However, as is the case in several other African countries, neonatal GBS colonization in Ethiopia has not been well documented. In addition, no preventive strategies for GBS infection have been yet formulated in the study area. Thus, this study was aimed to determine the prevalence of newborn colonization with GBS, its antibiotic susceptibility profile, and associated risk factors in University of Gondar referral hospital, Northwest Ethiopia.

## Methods

### Study area

The study was conducted at the University of Gondar Referral Hospital, Northwest Ethiopia. The University of Gondar Referral Hospital is one of the oldest hospitals located 737 km away from Addis Ababa, the Capital of Ethiopia with the Latitude of 12^o^31`N,and Longitude 37^o^25`E.. The Central Statistical Agency of Ethiopia population projection report and the Amhara National Regional State Health Bureau report showed that the Amhara region has a population of 20,018,988, of which, 49.92% were females, and 15.62% of the total population was urban inhabitants. The hospital serves about five million people. It has 450 to 600 delivery admission services a month. No GBS screening and provision of intrapartum antibiotic prophylaxis for pregnant women established yet in the hospital.

### Study Design and Period

A prospective cross-sectional study design was conducted between December 2016 and November 2017.

### Population

#### Source population

All newborns who were delivered at the University of Gondar Referral Hospital in Northwest Ethiopia were the source population.

#### Study population

The study populations were those newborns delivered from pregnant women whose gestational age was ≥ 35 weeks.

### Inclusion and exclusion criteria

#### Inclusion criteria

Newborns whose mothers not on antibiotics during delivery and those newborns who have been delivered vaginally at ≥35 gestational weeks of pregnancy, and infants ≤ 30 minutes were included in the study.

#### Exclusion criteria

Newborns whose mothers; did use vaginal cream, lubricants or traditional sterilizer (vinegar) in the last 10 days prior to giving birth; were in emergency room, severely ill, current vaginal bleeding, use of an intra-vaginal product in the past 24hours (douche, antifungal products), mentally unstable pregnant women; those who were in multiple birth and refusal for study participation from mothers or guardians were excluded.

### Sample size determination

The sample size was calculated using the single population proportion estimation formula by taking 5% as the prevalence of neonatal GBS colonization [19].

$n=\frac{{z^2}_{\alpha /2}\times p\left(1-P\right)}{d^2}$ Where; *n*= sample size, *p* = prevalence of neonatal colonization with GBS in Ethiopia (*p* = 5%), d= maximum allowable error (margin of error) = 0.05, *Z* = value of standard normal distribution (Z-statistic) at 95% confidence level (*z*=1.96) and it became 73 newborns; however, to increase the precision/validity of the findings, the sample size was increased to 385 by taking *p* = 50%.

### Variables

#### Dependent variable

Colonization of newborns with Group B Streptococcus (GBS), Antibiotic susceptibility patterns of GBS.

#### Independent variables

Maternal age, residence, education, and occupation, gestational age, parity, history of still birth, history of abortion, gravidity, antenatal care (ANC) visit, contraceptive use, history of preterm delivery, length of premature rupture of membrane (ROM), human immune deficiency virus (HIV) status, sex of newborn, Appearance, Pulse, Grimace, Activity, and Respiration (APGAR) score, history of neonatal death, newborn`s weight (Kg), resuscitation required, Newborn to mother immediate close contact (baby with the mother soon following delivery or baby not in the neonatal intensive care unit), duration of labor (hours).

#### Data collection, sampling technique and laboratory procedures

Demographic and biological data were collected from the newborns immediately following birth pregnant women with ≥ 35 gestational weeks of pregnancy by trained midwives at the maternity ward in the hospital until the pre-determined sample size was reached.

#### Questionnaire

A pre-tested questionnaire (Additional file 1) 5% (20) was used to collect the data for the assessment of the study participants` (pregnant women with ≥ 35 gestational weeks) demographic situations and to investigate the associated risk factors to newborn GBS colonization. Questionnaire were prepared in English using published studies with certain change and translated into the local language (Amharic). The response of each participant re-translated into English for analysis and report.

#### Biological Specimen collection

Three body surface site (nasal, umbilical and ear) swabs of newborns were collected and analyzed at the University of Gondar Microbiology Laboratory by using the recommended methods [10, 20].

#### Swab culture

Using the Centers for Disease Control and prevention (CDC) guidelines, nasal, umbilical and ear swabs were collected from each newborn and placed in the non nutritive Amies transport medium. Within 2 to 4 hours of collection, the swabs were placed in Todd-Hewitt selective enrichment broth supplemented with colistin (10μg/ml) and naldixic acid (15μg/mL) (Cart Roth GmbH + Co. KG-Schoemperlensrr. 3-5-D-76185 Karisruhe, Germany). The inoculated selective medium was incubated at 37 °C in 5% CO_2_ for 24hours. The growth (turbidity) was sub-cultured in 5% defibrinated sheep-blood agar and incubated for 24 hours at 37 °C in 5% CO_2_ atmosphere. All suspected colonies (with narrow hemplysis) were sub-cultured on nutrient agar and subjected to gram stain and catalase test. All gram positive cocci and catalase negative isolates were tested for CAMP factor for presumptive identification.

#### CAMP (Christie–Atkins–Munch-Petersen) test

CAMP test was used to differentiate GBS (CAMP positive) from *Streptococcus pyogene* (beta-hemolytic CAMP negative) by inoculating the known *Staphylococcus aureus* onto 5% defibrinated sheep blood agar down the center of the plate with a wire loop. Group B Streptococcus (test bacterium) was then streaked in a straight line perpendicular to the *S. aureus* within 2mm far. The plate was then incubated at 35 °C for 24 hours. A positive CAMP result was indicated by an arrowhead-shaped enhanced zone of beta-hemolysis in the area between the test organism and *S. aureus* with the arrow-point towards the *S. aureus* streak. The CAMP test positive colonies were presumptively considered as GBS

#### Antibiotic susceptibility testing of Group B Streptococcus

Susceptibility of GBS isolates were tested against 10 antibiotics (Oxoid, Basingstoke, UK):penicillin G (P, 10 IU), ampicillin (AMP, 10μg), clindamycin (CLY, 2μg), erythromycin (E, 15μg), chloramphenicol (C,30μg), ciprofloxacin (CIP,5μg), ceftriaxone (CRO, 30μg), vancomycin (VA, 30μg), Azithromycin (AZM, 15 μg), and tetracycline (TE, 30 μg) on Mueller-Hinton agar (MHA) containing 5% sheep blood according to the Kirby-Bauer method (disk diffusion) and the CLSI criteria. An inoculum was ready by suspending 4 -5 freshly grown GBS colonies in 3-5 ml sterile physiological saline. The turbidity was adjusted to a 0.5 McFarland standard [20, 21] used as a reference to adjust the bacterial suspension for antibiotic susceptibility test. The suspension was then swabbed over the entire surface of the Muller Hinton agar containing 5% defibrinated sheep blood by using sterile cotton tip applicator. Antibiotics disks were placed in the plate and incubated in 5% CO_2_ atmosphere at 37 °C for 24 hours. Zone of inhibition around antibiotic disks was measured by calibrated ruler and interpreted as sensitive, intermediate or resistant by comparing it with the standard chart [20].

#### Double disc diffusion

Clindamycin and erythromycin susceptibility tests and determination of different phenotypes of macrolide-lincosamide-streptogramin B (MLS_B_) resistance were performed by the double-disk test on Mueller-Hinton agar (Biokar, France) containing 5% sheep blood as previously described [20, 22–24]. Erythromycin (15 μg) and clindamycin (2 μg) disks (Oxoid, UK) were placed 12mm apart edge to edge [20]. After 24 hours of incubation at 37°C, blunting of the clindamycin inhibition zone proximal to the erythromycin disk was taken as inducible clindamycin resistance. Constitutive clindamycin resistance was the resistance to both clindamycin and erythromycin without blunting of the clindamycin inhibition zone. Susceptibility to clindamycin but resistance to erythromycin without blunting of the inhibition zone around the clindamycin disk was the efflux mechanism (the M-phenotype). Eventually, resistance to clindamycin but susceptible to erythromycin was referred to as L phenotype as previously described [24, 25].

#### Quality control

Half day training was given to the data collectors and they were closely supervised during data collection. Pre-test was done before the actual work to check the protocol for isolation of GBS and the questionnaire for collection of demography and clinical factors of the study participants. Data cleaning were done daily. *Streptococcus agalactiae* (ATCC 12386), *Enterococcus faecalis* (ATCC 29212); *Streptococcus pyogenes* (ATCC 19615), *Staphylococcus aureus* (ATCC 29213) and *Escherichia coli* (ATCC 25922) were used as quality control throughout the study.

#### Data analysis and interpretation

A total of 385 newborns were enrolled in the study and the collected data were entered into excel spread sheet and exported to SPSS 20 (Chicago, IL, USA) and analyzed. Association between the outcome variable (colonization of newborns with GBS) and each independent variable (demography and clinical factors) was analyzed using bi-variable and multi-variable logistic regression model. All the variables were entered into the multivariable logistic regression using backward LR method to control the confounding effect. Explanatory variables which had significant association with the newborn GBS colonization at a *p*-value ≤ 0.2 in the bivariable binary logistic regression model were entered to the multivariable logistic regression model to identify the factors associated to the colonization of newborns with GBS. Association between the outcome and the independent variables was calculated by using the adjusted odds ratio at a *p*-value ≤ 0.05 and 95% confidence interval. Assumption of goodness of the model was checked by Hosmer-lemeshow test (*p* = 0.828).

#### Ethical considerations

The study was reviewed and approved by the Ethical Review Committees of the University of Gondar (IRB) before data collection. Permission was obtained from the Hospitals administrative bodies. The study participants were informed about the study before collecting any data or samples. Written informed consent and/or assent obtained from the study participants. Ear, nasal and umbilical swabs were collected by experienced midwives and processed in the bacteriology laboratory using conventional methods. Participants (mothers) had full right to continue or withdraw their newborns from the study. Confidentiality of all participants’ information was maintained throughout the study.

## Results

### Demographic, obstetric characteristics and Group B Streptococcus colonization of newborns

As shown in Table 1, among the total of 385 newborns tested, 56.1% were males, 99.2% were delivered at > 37 gestational weeks of pregnancy, 89.6% newborns were weighed 2.5kg or more, and 82.1% of the newborn were delivered within 12 hours of labor. Most of the newborns` mothers (74.3%) were housewives and 35.6% of the mothers had secondary educational status followed by primary school level (34%).

**Table 1:** Newborns GBS colonization by demographic and obstetrics characteristics including multivariable analysis, Northwest Ethiopia

Characteristics	Response	GBS+	GBS-	COR^a^; 95% CI	AOR^b^, 95% CI^c^	*p-value*
Maternal age (yrs) Median = 25	<25	48	249	1	-	-
	≥25	14	74	1.019 (0.532, 1.951)	-	-
Maternal Residence	Urban	51	268	1	-	-
	Rural	11	55	0.951 (0.466, 1.942)	-	-
Maternal education	None	15	72	1.200 (0.418, 3.441)	4.800 (2.752, 8.372)	0.000004
	Primary	13	118	2.269 (0.784, 6.565)	8.371 (4.701, 14.909)	0.000004
	Secondary	28	109	0.976 (0.363, 2.609)	2.928 (1.851, 4.630)	0.000004
	Tertiary	6	24	1	1	
Maternal occupation	House wife	46	240	1	1	
	Employed	13	61	0.899 (0.457, 1.769)	2.244 (1.162, 4.331)	0.016
	Others	3	22	1.406 (0.404, 4.890)	2.102 (0.587, 7.530)	0.254
Gestational Age	<37wks	1	2	0.681 (0.061, 7.590)	NA	NA
	> /=37wks	61	321	1	NA	NA
Parity	multipara	32	164	1	1	
	nulipara	30	159	0.967 (0.561, 1.666)	3.641 (2.320, 5.714)	0.000
History of still birth	No	59	308	1	-	-
	Yes	3	15	0.958 (0.269, 3.412)	-	-
History of abortion	No	58	294	1	-	-
	Yes	4	29	1.430 (0.484, 4.223)	-	-
History of neonatal death	No	60	316	1	-	-
	Yes	2	7	0.665 (0.135, 3.277)	-	-
Gravidity	Primigravida	28	156	1	1	
	Multigravida	34	167	0.882 (0.511, 1.522)	3.507 (2.296, 5.355)	0.000
ANC visit	0 - 3	16	108	1.444 (0.782, 2.669)	-	-
	4 - 5	46	215	1	-	-
Contraceptive use	No	7	61	1	-	-
	Yes	55	262	0.547 (0.237, 1.259)	-	-
History of preterm delivery	No	60	317	1	-	-
	Yes	2	4	0.379 (0.068, 2.113)	-	-
Length of Premature ROM	≤1hr	48	223	1	-	-
	>1hr	14	100	1.537 (0.810, 2.917)	-	-
HIV status	No	59	313	1	-	-
	Yes	3	10	0.628 (0.168, 2.352)	-	-
Sex of newborn	Male	35	181	0.983 (0.568, 1.701)	-	-
	Female	27	142	1	-	-
APGAR^d^ Score at 1 minute	<7	5	50	2.088 (0.797, 5.467)	-	-
	7 - 10	57	273	1	-	-
APGAR Score at 5 minutes	<7	2	28	2.847 (0.661, 12.275)	-	-
	7 - 10	60	295	1	-	-
Newborn`s weight (Kg) median =3.0	<2.5	6	34	1.098 (0.440, 2.738)	-	-
	≥2.5	56	289	1	-	-
	Yes	16	307	1	-	-
Resuscitation required	No	58	287	1	1	
	Yes	4	36	1.819 (0.623, 5.307)	3.982 (1.113, 14.239)	0.034
Newborn to mother immediate close contact	No	12	76	1.282 (0.649, 2.532)	4.219 (3.058, 5.823)	0.000
	Yes	50	247	1	1	
Duration of labor(hour)	4 - 12	54	262	1	-	-
	13 - 24	8	61	1.572 (0.711, 3.473)	-	-

^a^crude odds ratio, ^b^adjusted odds ratio, ^c^confidence interval, ^d^Appearance, Pulse, Grimace, Activity, and Respiration

A total of 1,155 swabs from three body surface sites were collected and 81 (7.0%) of the specimens were positive for GBS. Among the newborns participated in this study, 62 (16.1%; 95% CI: 12.2-20.0) newborns were colonized with GBS and 56.5% of the GBS positive newborns were males. Among the newborns positive for GBS, 77.1% were delivered from those mothers whose age was < 25 years old.

A multivariable logistic analysis indicated that the newborns who were born to mothers whose educational status was below tertiary level; none (AOR = 4.800, 95% CI: 2.752, 8.372), primary (AOR = 8.371, 95% CI: 4.701, 14.909), and secondary (AOR = 2.928, 95% CI: 1.851, 4.630); were associated with an increased risk of colonization of newborns with GBS. Some of the maternal factors such as being employed (AOR = 2.244, CI: 1.162, 4.331), being nullipara (AOR = 3.641, 95% CI: 2.320, 5.714) and being multigravida (AOR = 3.507, 95% CI: 2.296, 5.355) were also at risk for newborn colonization with GBS. Moreover, we found that two neonatal factors, for instance, newborns who were in need of resuscitation (AOR = 3.982, 95% CI: 1.113, 14.239) and those newborns who did not have immediate contact (baby not with the mother soon following delivery or baby in the neonatal intensive care unit) with their mothers (AOR = 4.219, 95% CI: 3.058, 5.823) were associated with increased risk of newborns being colonized with GBS (Table 1).

### Colonization of newborns with Group B Streptococcus by the body surface sites

As noted above, 16.1% of the total newborns tested in this study were GBS colonized and of the total swabs processed, 81/1,155 (7.01%) were positive for GBS. Among the three newborn body surface sites swabbed, the nasal swabs accounted for more (8.1%, 95% CI: 5.2-11.3) colonization followed by the umbilical surface swabs (7.5%, 95% CI: 5.1-10.6). Fourteen (22.6%) of the newborns colonized with GBS in this study had more than one body surface site colonization, and 8.1% had three body surface site GBS colonization (Table 2).

**Table 2:** Newborns GBS* colonization by their body surface sites, Northwest Ethiopia (*n* = 62)

Newborn body site colonized	No. of GBS positive	Percentage (%)
Nasal swab^a^	31	8.1
Ear swab^a^	21	5.5
Umbilicus swab^a^	29	7.5
Total	62	16.1
Nasal swab only	19	30.6
Ear swab only	14	22.6
Umbilicus swab only	15	24.2
Nasal and ear swabs	0	0.0
Nasal and umbilicus swabs	7	11.3
Ear and umbilicus swabs	2	3.2
Nasal , ear and umbilicus	5	8.1
Total	62	100.0

*Group B Streptococcus/*Streptococcus agalactiae,* ^*a*^*overall prevalence (62; 16.1%) without combining body sites*

### Antibiotic susceptibility patterns of Group B Streptococcus isolates

All the isolates were tested for 10 commonly prescribed antibiotics by using the recommended methods. Of the GBS identified from the different body surface sites of the newborns, the antibiotics susceptibility ratess (an average of the three body sites tested) were 95.1%, 89.6%, 88.9%, 85.7%, 85.3%, 81.3%, 76.9%, 76.1%, 73.8%, and 34.4% to ampicillin, penicillin, ciprofloxacin, chloramphenicol, vancomycin, azitromycin, erythromycin, clindamycin, ceftriaxone, and tetracycline respectively (Table 3). The least susceptibility rate (average of the three body surface sites) was reported in tetracycline (34.3%). Ciprofloxacin, chloramphenicol, vancomycin and azithromycin were found to be the drug of choice next to ampicillin and penicillin in our study.

**Table 3:** Antibiotic susceptibility patterns of GBS isolated from the newborns` body surfaces, Northwest Ethiopia

Antibiotics	Disc potency		Colonizing GBS isolates		
		Newborn`s body sites	Susceptible, n(%)	Intermediate, n(%)	Resistant, n(%)
Penicillin	10units	Nasal nare (*n*=31)	29 (93.5)	0 (0.0)	2 (6.5)
		Umbilicus (*n*=29)	26 (89.7)	0 (0.0)	3 (10.3)
		Ear (*n*=21)	18 (85.7)	0 (0.0)	3 (14.3)
Ampicillin	10μg	Nasal nare (*n*=31)	29 (93.5)	0 (0.0)	2 (6.5)
		Umbilicus (*n*=29)	28 (96.6)	0 (0.0)	1 (3.4)
		Ear (*n*=21)	20 (95.2)	0 (0.0)	1 (4.8)
Erythromycin	15μg	Nasal nare (*n*=31)	24 (77.4)	2 (6.5)	5 (16.1)
		Umbilicus (*n*=29)	21 (72.4)	1 (3.4)	7 (24.2)
		Ear (*n*=21)	17 (80.9)	0 (0.0)	4 (19.1)
Clindamycin	2μg	Nasal nare (*n*=31)	24 (77.4)	1 (3.2)	6 (19.4)^a^
		Umbilicus (*n*=29)	22 (75.9)	2 (6.9)	5 (17.2)^a^
		Ear (*n*=21)	16 (76.2)	2 (9.5)	3 (14.3)^a^
Azitromycin	15μg	Nasal nare (*n*=31)	27 (87.1)	1 (3.2)	3 (9.7)
		Umbilicus (*n*=29)	22 (75.9)	4 (13.8)	3 (10.3)
		Ear (*n*=21)	17 (80.9)	3 (14.3)	1 (4.8)
Vancomycin	30μg	Nasal nare (*n*=31)	26 (83.9)	0 (0.0)	5 (16.1)
		Umbilicus (*n*=29 )	25 (86.2)	0 (0.0)	4 (13.8)
		Ear (*n*=21)	18 (85.7)	0 (0.0)	3 (14.3)
Ceftriazone	30μg	Nasal nare (*n*=31)	23 (74.2)	0 (0.0)	8 (25.8)
		Umbilicus (*n*=29)	22 (75.9)	0 (0.0)	7 (24.1)
		Ear (*n*=21)	15 (71.4)	0 (0.0)	6 (28.6)
Ciprofloxacin	5μg	Nasal nare (*n*=31)	29 (93.5)	0 (0.0)	2 (6.5)
		Umbilicus (*n*=29)	24 (82.8)	0 (0.0)	5 (17.2)
		Ear (*n*=21)	19 (90.5)	0 (0.0)	2 (9.5)
Chloramphenicol	30μg	Nasal nare (*n*=31)	26 (83.9)	3 (9.6)	2 (6.5)
		Umbilicus (*n*=29)	24 (82.8)	3 (10.3)	2 (6.9)
		Ear (*n*=21)	19 (90.4)	1 (4.8)	1 (4.8)
Tetracycline	30μg	Nasal nare (*n*=31)	8 (25.8)	3 (9.7)	20 (64.5)
		Umbilicus (*n*=29)	10 (34.5)	3 (10.3)	16 (55.2)
		Ear (*n*=21)	9 (42.9)	0 (0.0)	12 (57.1)

^a^Excluding the inducible clindamycin resistant isolates (iMLSB)

### Inducible and constitutive resistance Group B Streptococcus isolates to clindamycin

**Fig. 1:**
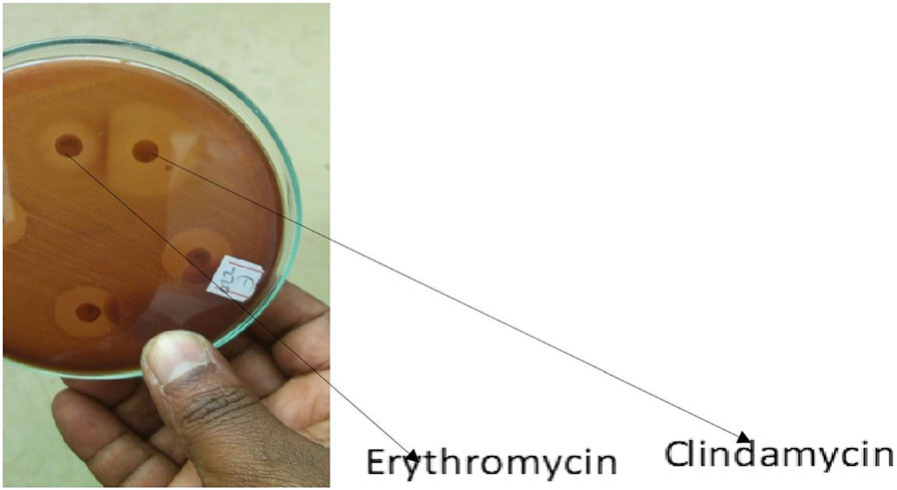
Inducible Clindamycin resistance (D-zone) of GBS isolated from a newborn ear swab, Northwest Ethiopia. Legend: A *S. agalactiae* isolated from a newborn ear swab from the University of Gondar referral hospital in Northwest Ethiopia showed Inducible MLS_B_ phenotype (erythromycin-resistant and clindamycin sensitive) determined by means of an antibiotic disk diffusion test or D-test (the blunting of the clear circular area of no growth around the *clindamycin* disk on the side adjacent to the erythromycin disk and was designated as D-test positive)

The phenotypic analysis of GBS isolates identified from the three body surfaces sites of the newborns was done by using erythromycin and clindamycin double disc diffusion (D-zone testing) method as per the CLSI 2017 guideline. Among the 32 GBS isolates resistant and/or intermediate resistant to erythromycin and clindmaycin, 34.4% harboured L phenotype, 31.3% had M phenotype, 21.9% had constitutive Macrolide, Lincosamide-Streptogramin (B) (cMLSB) and 12.5% contained inducible Macrolide, Lincosamide-StreptograminB (iMLSB) (Fig. 1 Legend, and Table 4). We found 12.5% inducible and 21.9% constitutive resistance GBS to clindamycin.

**Table 4:** Macrolide, Lincosamide-StreptograminB (MLSB) and D-shape of the GBS isolates in Northwest Ethiopia

Double disc diffusion^a^								
GBS phenotypes	Erythromycin (n)			Clindamycin (n)			Total (*n* = 32)	Percent (%)
	R, </=15mm	I, 16-20mm	S, >/=21mm	R,</=15mm	I,16-18mm	S,>/=19mm		
constitutive macrolide, lincosmide-streptogramin B (cMLSB)	7	0	-	6	1	-	7	21.9
inducible macrolide, lincosmide-streptogramin B (iMLSB)	4	0	-	0	0	4	4	12.5
M-phenotype	6	4	-	-	-	10	10	31.3
L-Phenotype	-	-	11	7	4	-	11	34.4
D – Shape positive	4	0	0	-	-	4	4	12.5
D – Shape negative	-	-	-	-	-	-	28	87.5

^a^CLSI (2017) disk diffusion breakpoints [57]. For erythromycin: ≥21 mm, susceptible (S); 16 to 20 mm, intermediate (I); ≤15 mm, resistant (R). For clindamycin: ≥19 mm, susceptible (S); 16 to 18 mm, intermediate (I); ≤15 mm, resistant (R)

## Discussion

Our study showed that 62 (16.1%; 95% CI: 12.2-20.0) of the newborns participated in the study were colonized with GBS, which could be the possible causes to the high morbidity and mortality of neonates in the study area. This prevalence of colonization was in agreement with different studies conducted worldwide such as: France (13.9%) [26], Turkey (17.3%) [27]), South Africa (15.8%) [14] and Gambia (12.0%) [28]. Contrary to our study, other studies showed the lower prevalence of newborn colonization with GBS and some of these were Iran (1.7% to 5.5%) [29–31], Saudi Arabia (1.0%) [32], Turkey (1.6% to 8.0%) [12, 33], Pakistan (6.0%) [13], China (4.9%) [34], India (1.3% to 3.2%) [35, 36], Korea (1.5%) [37], Bangladesh (6.3% to 7.4%; in which, the finding from umbilicus is in agreement with ours) [38, 39], Lithuania (6.4%; where 5.3% GBS were isolated from the ear swab of the newborns as it was observed in our study and 4.6% from the throat) [40], Greek (2.4%) [41], Nigeria (6.8%) [42], Tanzania (8.9%) [43] and Ethiopia (5%) [19]. The discrepancies might be associated with the Global variability of maternal colonization with GBS (differences in geography, season, IAP provision), the mode of delivery (in which newborns born by spontaneous vaginal delivery had usually more GBS colonization), and the availability of laboratory facilities and experiences of laboratories to detect GBS.

Inconsistent to our result, a lot of studies showed higher neonatal colonization with GBS, for example, studies in Poland (26.7% to 34.5%) [44, 45] and Bangladesh (38%) [39]. The regional differences, variability in the sample size, methods employed for GBS detection, availabilities of laboratory facilities, experiences of laboratory technologists, newborn body surface sites swabbed and time of sample collection (soon after birth or later) might be possibly explained the disparities. The differences could also be explained by the presence or absence of the IAP administration, variations of maternal colonization and density of GBS colony and mode of delivery.

In our study, the antibiotics susceptibility rates of GBS were 95.1%, 89.6%, 88.9%, 85.7%, 85.3%, 81.3%, 76.9%, 76.1%, 73.8%, and 34.4% to ampicillin, penicillin, ciprofloxacin, chloramphenicol, vancomycin, azitromycin, erythromycin, clindamycin, ceftriaxone, and tetracycline respectively. We identified that ciprofloxacin, chloramphenicol, vancomycin and azithromycin were the drug of choice next to ampicillin and penicillin. The GBS in the current study showed better sensitivity to azithromycin than erythromycin and clindamycin. Thus, given the recent interest in the azithromycin, it is wise to do more study on this drug and consider it as the alternative prophylaxis for the penicillin allergic laboring mothers to reduce the carriage of GBS in mothers and newborns and then to lower the risk of neonatal diseases beyond the trachoma control.

In agreement with our findings, a study in Egypt showed that 29.4% of the GBS isolated from the neonates were resistance to erythromycin and 17.6% were resistance to clindamycin [46]. Another studies conducted in different parts of the world such as, in France showed that 25.6% were resistance to clindamycin and 38.2% to erythromycin [18] and 21.4% to macrolide [47], in the USA, 20.2% to 32% were resistance to erythromycin and 6.9% to 15% to clindamycin [17, 48, 49], and in Italy, 17% were resistance to erythromycin and 15.3 % to clindamycin [50]. Another report from Tanzania revealed that the neonatal GBS were 100% susceptible to penicillin, ampicillin, vancomycin and ciprofloxacin whereas susceptibility to ceftriaxone, clindamycin and erythromycin were 93.8%, 87.5% and 81.3% respectively [43]. A study in Germany also reported that all the isolates were susceptible to beta-lactams and vancomycin while 10.1% were resistance to erythromycin and 5.7% to clindamycin [51] which are lower than our reports. This variation might be explained by the fact that the laboratory facilities and health literacy of the people in our setting are different from other developed countries.

Contrasting to the results of our study, a Chinese report revealed that all the GBS isolated from the neonates were susceptible to penicillin, but the rates of resistance to clindamycin and erythromycin were 84.0% and 88.0% [52]. These discrepancies may dictate that the rates of resistance to erythromycin and clindamycin varied among geographic regions and were notably the highest in China. Additionally, a study explained that antibiotics currently prevent an estimated 29,000 cases of early onset GBS disease per year. This approach may challenge in the low-income countries where many births take place at home, and laboratory capacity for the screening of GBS is limited [8]. The provision of antibiotics to pregnant women without screening may also contribute to the emergence of antibiotic resistance. An alternative prophylaxis failure is becoming more likely to the increasing of macrolide resistance rates among the GBS isolates. Therefore, in cases when considering these antibiotics, including azithromycin as alternatives for prophylaxis and treatment for GBS, susceptibility test should be done before the prescriptions. We also reported that among the GBS tested for MLS_B_ by using the double disk diffusion technique, 34.4%, 31.3%, 21.9% and 12.5% isolates had L- and M- phenotypes, cMLS_B_ and iMLS_B_, respectively. The inducible and constitutive resistance reported in our study is lower than a study from Canada, where 40.0% of the isolates were inducible and 47.3% were constitutive resistance to clindamycin [24].

Of the possible factors associated for the colonization of the newborns that were investigated in our study, the newborns born to mothers whose education status was below tertiary level and from employed mothers had the increased risk of colonization with GBS. It could be justified by the fact that keeping personal hygiene is likely better among those people who have more education status than their counterparts. In addition, employment may increase mobility of women and expose them for different causal partnerships with different people who could be the risks for them, later becomes a source for their neonatal colonization. Being nulliparity and multigravida are among the maternal factors, and newborns who were in resuscitation and who didn`t have immediate contact with their mothers (in the neonatal intensive care unit) had also the increased risk of newborn colonization.

Congruently, different literatures presented that women with less (or no formal,/lack of) education, women of lower parity, multigravid, young maternal age (< 20 yrs), vaginal mode of delivery, intrapartum fever, prolonged premature rupture of membrane, preterm gestational age, low birth weight (< 2.5kg), and neonatal intensive care admission were associated with neonatal colonization with GBS [43, 46, 53–56]. Likewise, a study in Tanzania showed that prolonged duration of labour had the significant association with colonization of the newborns with GBS, possibly due to the extended exposure of the newborns in the birth canal [43]. This calls for the screening of pregnant women for GBS at their 35 to 37 weeks of pregnancy and provision of IAP for those women who have been positive for GBS to reduce the chances of later neonatal colonization.

We found that maternal age, obstetric history, gestational age, sex of the newborn, HIV infection, Appearance, Pulse, Grimace, Activity, and Respiration (APGAR) score, preterm delivery, number of antenatal care (ANC) visit, and duration of labour did not show a significant association with neonatal colonization. In agreement to this, Joachim and his co-workers in Tanzania [43] presented that prolonged premature rupture of membrane, intrapartum fever, mode of delivery and low birth weight did not influence neonatal colonization with GBS. Tsolia et al. [41] in their study reported that the multiparity (≥2 previous births) is associated with a low risk for maternal colonization with GBS. It might be explained by the numbers of participants in our study with these risk factors were small. It is useful to know that GBS could be transferred from pregnant women to newborns, and was evidenced by the fact that after the Caesarean section was done (before rupture of the membrane), molecular strain identification demonstrated that same GBS strain was found in mothers and their newborns [45]. So to prevent neonatal colonization with GBS and to increase newborn health conditions, prevention strategies should be developed and promoted in the study area.

### Limitations

This study has main limitations in terms of small sample size, non-probability sampling method, and using only disc diffusion for antibiotic susceptibility test.

## Conclusion

Prevalence of newborn colonization with GBS in this study was higher than the findings reported three Decades ago in the same area. We identified that ciprofloxacin, chloramphenicol, vancomycin and azithromycin were the drug of choice next to ampicillin and penicillin. In addition, 12.5% of the isolates in our study showed inducible clindamycin resistance. Lower education status, being employed, and being nullipara and multigrapvida were the maternal factors associated with the increased risk of newborn colonization. Resuscitation and denial of the newborn`s immediate contact with their mothers were the neonatal factors which showed the increased risk of newborn colonization. So to prevent neonatal colonization with GBS, continuous health education, screening of pregnancy for GBS at the 35 to 37 weeks of gestation and provision of IAP for those positive cases for GBS should be promoted in the study area. GBS surveillance and their antibiotic susceptibility testing should also be conducted in the country by using advanced laboratory technologies.

## Additional file

Additional file 1: Questionnaire for Newborn colonization with GBS, University of Gondar Referral Hospital, Northwest Ethiopia (DOCX 29 kb)

## Abbreviations

ANC: Antenatal Care
AOR: Adjusted Odds Ratio
APGAR: Appearance, Pulse, Grimace, Activity, and Respiration
ATCC: American Type Culture Collection
CAMP: Christie–Atkins–Munch-Petersen
CI: Confidence Interval
CLSI: Clinical Laboratory Standard Institute
CO_2_: Carbon dioxide
GBS: Group B Streptococcus
HIV: Human Immune deficiency Virus
IAP: Intrapartum Antibiotics Prophylaxis
MLS_B_: Macrolide-Lincosamide-Streptogramin B
PROM: Premature Rupture of Membrane
*S. agalactiae*: *Streptococcus agalactiae*
*S. aureus*: *Staphylococcus aureus*
SPSS: Statistical Package for Social Sciences
μg/L: Microgram per liter
