# Supplementary material for: Newborn colonization and antibiotic susceptibility patterns of Streptococcus agalactiae at the University of Gondar Referral Hospital, Northwest Ethiopia
Source: BMC Pediatr. 2018 Nov 30;18:378. doi: 10.1186/s12887-018-1350-1 (PMC6271408; doi:10.1186/s12887-018-1350-1)
Supplement: Supplementary file 1 — Questionnaire for Newborn colonization with GBS, University of Gondar Referral Hospital, Northwest Ethiopia (DOCX 29 kb) [file 12887_2018_1350_MOESM1_ESM.docx]

**Supplement 1: Questionnaire for Newborn colonization with GBS, University of Gondar Referral Hospital, Northwest Ethiopia,**

**University of Gondar**

**College of Medicine and Health Sciences**

**Department of Medical Microbiology**

1. **Questionnaire prepared for studying GBS colonization in pregnant women and their newborns**
2. **Maternal details**

| **S/No.** | **Part I Hospital information ( Ask her whether she is on ANTIBIIOTICS CURRENTLY)** | |
| --- | --- | --- |
| 001 | Pregnant woman`s Serial No. | - __________________________________ |
| 002 | Pregnant woman`s | - Medical Registration Number_________________ - Study Code Number. _________________________ |
| 003 | Pregnant woman`s contact | - Tel Number ________________________ - Name of District_____________________ - Name of kebele_____________________ |
| 004 | Date Recruited: | - _______/_________/___________\|   (DD/ MM/ YY) |
| 005 | Name of Hospital | 1. Gondar 2. Felegehiwot 3. Debre-Markos 4. Dessie |
| 006 | Ward | 1. ANC 2. Obstetrics 3. Gynecology 4. Neonatology 5. Pediatrics |
|  | **Part II. Socio-demographic characteristics** | |
| 101 | Maternal age? | ______________Years |
| 102 | Place of residence? (Name it) | 1. Urban 2. Rural |
| 103 | Marital status? | 1. Married 2. Divorced 3. Widowed 4. Unmarried |
| 104 | Level of education? | 1. No education 2. Primary (1-8^th^ grade) 3. Secondary (9-12^th^ grade) 4. Tertiary |
| 105 | Current occupation? | 1. House wife 2. Government employee 3, Private employee 4. Farmer 5. Student 2. Business woman 7. Daily laborer 8. Others (specify)_____________________ |
| 106 | Husband`s/partner`s level of education? | 1. No education 2. Primary (1-8^th^ grade) 3. Secondary (9 -12^th^ grade) 4. Tertiary |
| 107 | Husband`s / Partner`s occupation | 1. Government employee 2. Private employee 3. Farmer 4. Student 5. Business man 2. Daily laborer 7. Others (specify)____________________________ |
|  | **Part III: Obstetric History** | |
| 201 | Gestational week of the current pregnancy? | ___________________weeks |
| 202 | Mode of previous delivery? | 1. SVD 2. Cesarean section 3. Instrumental assisted delivery 4. First pregnancy |
| 206 | History of stillbirth | 1. No 2. Yes |
| 207 | History of miscarriages /abortion | 1. No 2. Yes |
| 208 | History of neonatal deaths? | 1. No 2. Yes |
|  | **Part IV: Present Pregnancy** | |
| 301 | Number of Gravida | 1. One 2. Two 3. Three 4. Four 5. Five 6. Six 7. More than six |
| 302 | Number of ANC visit | 1. One time 2. Two times 3. Three times 4. Four times 5. More than 4 times |
| 303 | Have you ever used contraceptive? | 1. No 2. Yes |
| 304 | If yes to question No. 303, which contraceptive (s) used? **Multiple answer is possible** | 1. Oral contraceptive pills 2. Injectable (Depo Provera) 3. Implant (Implanton )for 3 years 2. Implant (Jadel) for 5 years 5. Loop (IUCD) 6. Condom |
| 305 | Meconium stained amniotic fluid | 1. No 2. Yes |
| 306 | If yes for question number 305, grade it. | 1. Grade one 2. Grade two 3. Grade three |
| 307 | Pre-term delivery? | 1. No 2. Yes |
| 308 | Any trauma during current pregnancy? | 1. No 2. Yes, **If yes (specify):_____________________________________________** |
| 309 | Presence of vaginal discharge that sought treatment during current pregnancy? | 1. No 2. Yes |
| 310 | ABO blood group? | 1. A 2. B 3. O 4. AB |
| 312 | Chronic illness during current pregnancy? | 1. No 2. Yes |
| 313 | If yes to question No. 312, what was a disease? | 1. Hypertension 2. DM 3. Kidney disease 4. Liver diseases 5. Cardiac disease   6. Asthma |
| 314 | Duration of rupture of membrane? | _______________hours |
| 315 | Intra-partum fever during current pregnancy? | 1. No 2. Yes , If yes, Temperature in ^O^C:_______________ |
| 318 | Chorioamniontis? | 1. No 2. Yes |
| 319 | HIV status? | 1. Negative 2. Positive |
| **320** | **Type of specimen needed** | 1. **Recto-vaginal swab** |
| 321 | Date & time of specimen collection |  |
|  | **Part V. Comment if any** |  |

1. **Details of the baby (newborn) from delivery to 90 days**

| 401 | Mother`s Serial No. | - ________________________________ |
| --- | --- | --- |
| 402 | Mother`s Medical Registration Number | - ________________________________ |
| 403 | Study Code number (**Neonate**) | - ______________________________ |
| 404 | Sex of newborn | 1. Male 2. Female |
| 405 | Age (in minute) of the newborn | ___________________days |
| 406 | Weight (in kg) of the newborn | ___________________Kgs |
| 407 | Stillbirth | 1. Fresh 2. Macerated |
| 408 | Alive | 1. No 2. Yes |
| 409 | Antibiotic / drug treatment of the newborn | 1. No 2. Yes, **If yes**, name the drug(s) ________________________________ |
| 410 | Congenital abnormality | 1. No 2. Yes, **If yes specify**:_________________________________________ |
| 411 | Fetal: APGAR**** score in: | 1. One minute 2. Five minutes |
| 412 | Resuscitation required | 1. No 2. Yes |
| 413 | Fever (body temperature) of the newborn | 1. No 2. Yes, **If yes**, record it in ^o^C___________________ |
| 414 | Length of hospital stay of the newborn | _______________hours |
| 415 | Close contact of the newborn with the mother (Is the newborn breast-feeding?) | 1. No 2. Yes |
| 416 | Digital vaginal examination after onset of labor or rupture of membrane (>5x) | 1. No 2. Yes |
| 417 | What was the duration of labour? | ______________hours |
| 418 | Did the mother has any of the health problem after delivery? | 1. No 2. Puerperal sepsis 3. Endometritis 4. UTI 5. Wound infection   6. Other (specify)______________________ |
| **419** | **Type of specimen needed** | - **Ear Swab** - **Umbilicus bases swab** - **Nasal nares swabs** |
| 420 | Date & time of specimen collection | - **_________________________________** |
| 421 | GBS disease developed by newborn (**to be filled after phone call or Medical chart or record review**) | 1. Sepsis 2. Pneumonia 3. Meningitis 4. Other (specify)___________________________  - Results of Laboratory investigation___________________________________ |

**-***Spontaneous vaginal delivery; ****** Group B Streptococcus**; ***** Early onset disease; ******** Appearance, Pulse, Grimace, Activity, Respiration

**Thank you very much for taking the time to complete our study!**

Name of data collector ______________________________________

Signature ________________ Date ______________
